# Immune Checkpoint Inhibitors in Cancer Treatment and Incidence of Pancreatitis

**DOI:** 10.7759/cureus.68043

**Published:** 2024-08-28

**Authors:** Oluchukwu Chimuanya Nwankwo, Francisco Martin Lara-Salazar, Santiago Lara-Salazar, Abdulrashid Onimisi Abdulrahim, Ijeoma Chijioke, Jyoti Singh, Ikhlaq Koradia, Nicole M Gomez, Rohit Prakash, Ragini Gopagoni, Megha Joshi, Manju Rai

**Affiliations:** 1 Internal Medicine, National Pirogov Memorial Medical University, Vinnytsia, UKR; 2 Internal Medicine, Universidad Autónoma de Guadalajara Facultad de Medicina Guadalajara, Zapopan, MEX; 3 Internal Medicine, Universidad de Guadalajara, Centro Universitario de Ciencias de la Salud, Guadalajara, MEX; 4 Internal Medicine, King Fahad Specialist Hospital, Buraidah, SAU; 5 Internal Medicine, Ross University School of Medicine, Bridgetown, BRB; 6 Surgery, King George's Medical College, Lucknow, IND; 7 Internal Medicine, Rajiv Gandhi Medical College, Thane, IND; 8 Medicine, Universidad Iberoamericana, Santo Domingo, DOM; 9 Orthopaedics and Trauma, Medway NHS Foundation Trust, Gillingham, GBR; 10 Internal Medicine, Malla Reddy institute of Medical Sciences, Hyderabad, IND; 11 Internal Medicine, Smt. Nathiba Hargovandas Lakhmichand Municipal Medical College, Ahmedabad, IND; 12 Biotechnology, Shri Venkateshwara University, Gajraula, IND

**Keywords:** pancreatitis pathophysiology, immune-related adverse events., pancreatitis, immunotherapy, immune checkpoint inhibitors

## Abstract

Immune checkpoint inhibitors (ICIs) are an approved therapy for the management of various advanced neoplasms. Limited reviews focus on the influence of this therapy resulting in pancreatitis. This review discusses the relationship between ICIs and their effects on the pancreas, including the incidence of pancreatitis, immunotherapy, programmed cell death 1 (PD-1) receptors, driver mutations, programmed death ligand 1 (PD-L1), and immune-related adverse events. Additionally, it focuses on the clinical presentations, diagnosis, case studies, and mechanisms by which ICIs activate different pathways to cause pancreatitis. We conducted a comprehensive literature search using PubMed, Cochrane Library, and Google Scholar databases to identify relevant studies on ICI-associated pancreatitis. The review explores the incidence and epidemiology of ICI-induced pancreatitis, its clinical presentation, diagnostic criteria, and management strategies.The overall incidence of ICI-induced pancreatitis is estimated at 1-2%, with higher rates observed in combination therapy. Clinical presentations range from asymptomatic enzyme elevations to severe pancreatitis. Diagnosis relies on a combination of clinical symptoms, elevated pancreatic enzymes, and imaging findings, with MRI and endoscopic ultrasound showing promise in early detection. Management strategies include IV fluid administration, pain control, and nutritional support. The efficacy of corticosteroids remains controversial, and alternative immunosuppressants are being explored for steroid-refractory cases. Long-term monitoring is crucial due to the risk of chronic pancreatitis and pancreatic insufficiency. This review highlights the need for further research to elucidate the exact mechanisms of ICI-associated pancreatic injury, develop predictive biomarkers, and refine treatment protocols. As ICI use continues to expand, a thorough understanding of this adverse event is essential for optimizing patient care and outcomes in cancer immunotherapy.

## Introduction and background

The landscape of cancer treatment has been revolutionized by the advent of immune checkpoint inhibitors (ICIs), a form of immunotherapy that has transformed patient outcomes worldwide. Traditional cancer management strategies, including chemotherapy, radiotherapy, surgery, and targeted therapy, while effective, often face challenges related to late presentation and diagnosis [1]. ICIs have emerged as a promising alternative, offering new hope for patients with various malignancies.

Immune checkpoints are physiological ligands that limit autoimmunity by suppressing potential autoreactive naive T cells in lymph nodes and deactivating T cells in peripheral tissues [2]. Tumor cells exploit these mechanisms to evade immune recognition. ICIs, distinct from other immunotherapy approaches like adoptive T cell therapies (e.g., chimeric antigen receptor T cell (CAR-T) therapy) and cancer vaccines, specifically target these checkpoint pathways.

The primary targets of ICI drugs are antibodies against immune inhibitory receptors: cytotoxic T lymphocyte antigen 4 (CTLA-4), programmed cell death 1 (PD-1), and programmed death ligand 1 (PD-L1). Nivolumab and pembrolizumab, examples of PD-1 inhibitors, have shown efficacy in treating various solid tumors [3]. Emerging research is also focusing on newer, less studied immune checkpoint receptors, such as lymphocyte gene 3 (LAG3) and T cell immunoglobulin and mucin protein (TIM3), which can be co-expressed on exhausted PD-1 T cells in the tumor microenvironment, promoting anti-tumor immune evasion [1].

Despite the promise of ICI therapy, only a subset of patients (20-40%) derive significant benefits, underscoring the need for predictive biomarkers [4]. For instance, the derived inflammation-linked neutrophil-to-lymphocyte ratio (dNLR) has shown potential in non-colorectal gastrointestinal (GI) cancer patients treated with ICIs, with higher levels (cut-off ≥ 3) correlating with worse outcomes [5].

**Figure 1:**
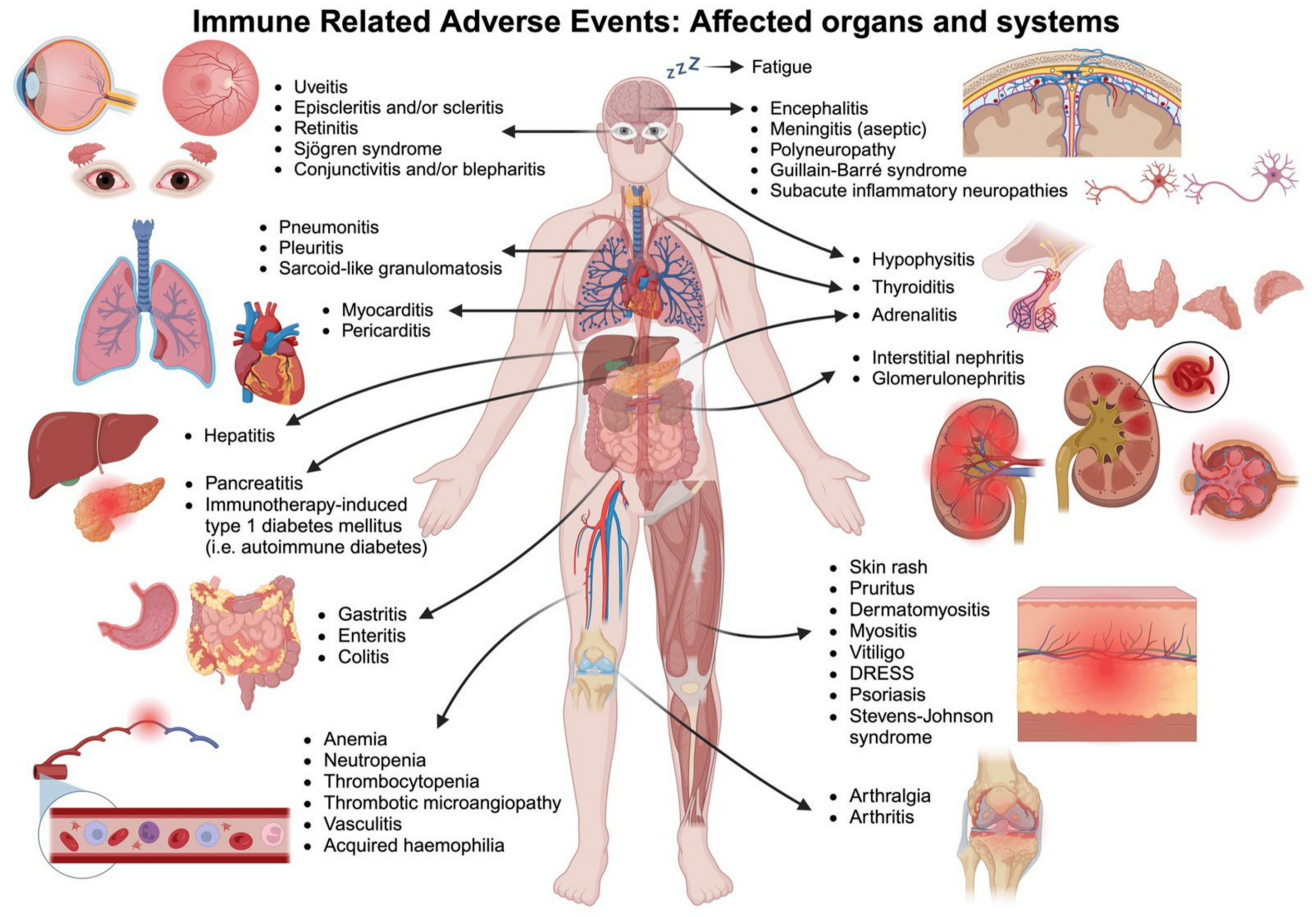
Immune-related adverse events: affected organs and systems The figure was created in BioRender.com.

While immune-related adverse events (irAEs), such as pruritus, diarrhea, and rashes, are more common, the incidence of pancreatitis in patients with ICIs is relatively low (Figure 1). However, immune-mediated pancreatitis presents unique challenges due to its variable clinical presentations, poor laboratory correlates, and elusive pathophysiology [6]. Studies have shown that PD-1 inhibitors carry a significantly higher risk of pancreatic adverse events (AEs) than PD-L1 inhibitors. Moreover, patients receiving dual ICI therapy face a higher risk of developing pancreatitis compared to those on single ICI therapy [7].

The timing and approach to dual immunotherapy administration also play a crucial role in the risk of ICI-induced pancreatic injury. Asynchronous administration of dual ICIs has been identified as a risk factor, while a higher incidence of grade ≥3 pancreatitis is associated with simultaneous rather than asynchronous dual ICI therapy [8]. It is important to note that ICI therapy can affect both endocrine and exocrine pancreatic function.

This review aims to explore the relationship between ICI therapy and the incidence of pancreatitis in cancer patients, assess its prevalence and severity, identify potential risk factors and mechanisms, evaluate current diagnostic and management strategies, and discuss implications for treatment continuation and cancer outcomes. By comprehensively examining the current literature, we seek to provide valuable insights for oncologists, gastroenterologists, and researchers, contributing to improved patient care and informed decision-making in cancer immunotherapy.

## Review

Methods

We conducted a comprehensive literature search to identify relevant studies on ICIs in cancer treatment and their association with pancreatitis. PubMed, Cochrane Library, and Google Scholar electronic databases were searched. The search was performed using various combinations of keywords and Medical Subject Headings (MeSH) terms, including but not limited to "immune checkpoint inhibitors," "cancer," "neoplasms," "pancreatitis," "CTLA-4," "PD-1," "PD-L1," "ipilimumab," "nivolumab," "pembrolizumab," and "adverse events."

The inclusion criteria for the review were original research articles, systematic reviews, meta-analyses, and case reports published in English. We focused on studies that reported on the use of ICIs in cancer treatment and either primarily investigated or reported cases of pancreatitis as an AE. There was no restriction on the publication date to ensure a comprehensive overview of the topic. Data extraction was performed using a standardized form, capturing information such as study design, sample size, types of ICIs used, cancer types, incidence of pancreatitis, severity grading, management strategies, and outcomes.

Overview of ICIs

ICIs have revolutionized cancer treatment over the past decade, significantly improving patients' quality of life and clinical outcomes [9]. These agents amplify the immune response against cancer cells by blocking inhibitory pathways that suppress T-lymphocyte activity, promoting sustained immune responses [9-10].

ICIs primarily target two major pathways: cytotoxic T-lymphocyte-associated protein 4 (CTLA-4) and PD-1 or its ligand PD-L110,11. CTLA-4, expressed on activated T cells, normally downregulates immune responses by inhibiting further T cell activation and expansion. By blocking CTLA-4, ICIs enhance T cell activation and proliferation, improving immune system function against cancer cells [9-11]. PD-1, expressed on multiple immune cell types, including T lymphocytes, B lymphocytes, and natural killer cells, inhibits T cell function when bound to its ligand PD-L1 [10-11]. PD-1 inhibitors prevent this suppression, promoting improved immune responses. Similarly, PD-L1 inhibitors bind to PD-L1, preventing its attachment to PD-1 and allowing T lymphocyte activity against tumors to proceed [10-11].

The mechanism of action of ICIs involves disrupting the delicate balance of immune system regulation. Normally, this balance prevents autoimmune conditions by maintaining an equilibrium of stimulatory and inhibitory signals [11]. Cancer cells exploit inhibitory pathways to escape immune surveillance. ICIs work by regulating these pathways, impeding their functions, and restoring the immune system's ability to recognize and attack cancer cells. Several ICIs have been approved for clinical use. Anti-CTLA-4 antibodies include ipilimumab (IgG1) and tremelimumab (IgG2) [9,12-13]. PD-1 inhibitors include nivolumab, pembrolizumab, and cemiplimab (all IgG4 mAbs) [12-14]. PD-L1 inhibitors include atezolizumab, durvalumab, and avelumab [13-14].

ICIs have demonstrated efficacy across various cancer types, particularly those previously difficult to treat [9]. CTLA-4 and PD-1 inhibitors are first-line treatments for metastatic melanoma, significantly improving patient survival [9-10,15]. PD-L1 inhibitors, alone or combined with chemotherapy, effectively treat non-small cell lung cancer (NSCLC), especially in patients with high PD-L1 expression [16]. For renal cell carcinoma, PD-1 and PD-L1 inhibitors have shown improved survival compared to conventional therapies [17]. Advanced or metastatic urothelial carcinoma is treated with PD-L1 inhibitors, particularly in patients unsuitable for standard chemotherapy [16]. PD-L1 inhibitors are also effective against recurrent or metastatic head and neck squamous cell carcinoma [11,16].

ICIs have expanded treatment options for various other cancers. Ipilimumab, combined with nivolumab, is approved for treating microsatellite instability-high (MSI-H) or mismatch repair (MMR) deficient metastatic colorectal cancer, intermediate- or poor-risk renal cell carcinoma, and hepatocellular carcinoma in patients previously treated with sorafenib [16]. The combination is also a first-line treatment for malignant pleural mesothelioma and NSCLC with PD-L1 expression ≥1% [16].

Despite their success, ICIs face challenges, including limited efficacy in some patients and the potential for irAEs. Ongoing research focuses on developing new ICIs, identifying predictive biomarkers, and optimizing combination therapies to enhance treatment efficacy while minimizing side effects [14,18].

Mechanism of pancreatitis induced by ICIs

Pathophysiology of Pancreatitis

Pancreatitis is the inflammation of the pancreas; this can be acute or chronic. The most widely known pathogenesis of pancreatitis involves the activation of the pancreatic digestive enzymes, causing a pro-inflammatory state in the pancreas, based on an abnormal intracellular activation of trypsinogen [19 ].

Furthermore, depending on the etiology, there is damage and injury to the pancreatic tissue, causing a release of damage-associated molecular patterns (DAMPs) causing a proinflammatory environment stimulating the activation of the innate immune system, intestinal permeability, and the translocation of gut microbiota and expression of genes related to inflammation and necroptosis, thus enhancing inflammation and activation of trypsinogen [19].

Immunological Basis for Immune AEs Induced by ICI

The ICI inactivates key regulators of the immune system, which are regulatory pathways that downregulate immune response to certain antigens; as a result, there is more powerful activation and priming of the adaptive immune system. Despite T cells having the main role in the mechanism of action of the ICIs, there is also a contribution of B cells, cytokines, and genetics to the therapeutic effect and the irAEs [20].

**Figure 2:**
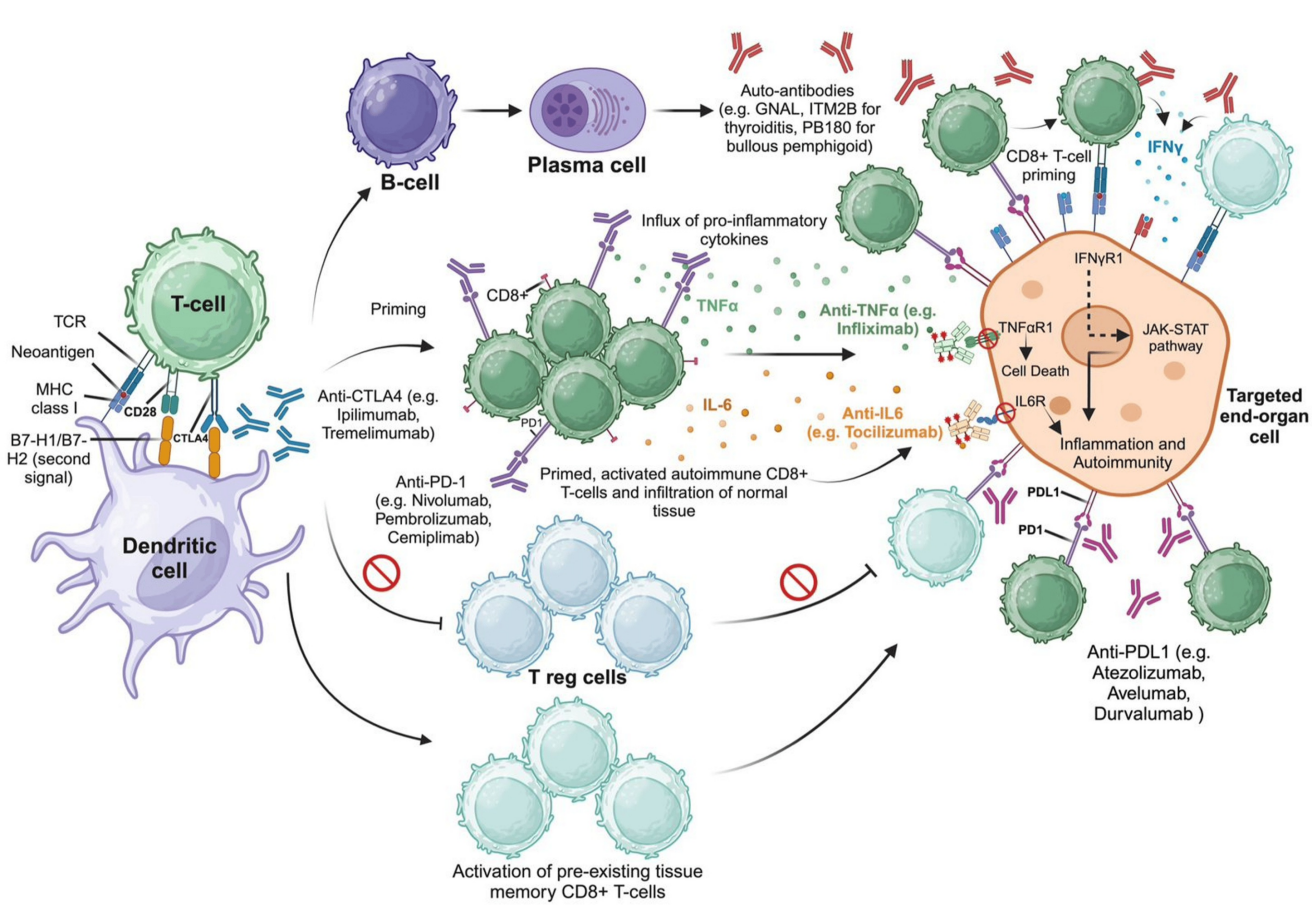
Immune checkpoint inhibition and immune-related adverse events: autoimmune microenvironment, mechanisms, and comprehensive therapeutic targets Figure created in BioRender.com.

The ICI toxicity works as an autoinflammatory and autoimmune disease (Figure 2). There are four mechanisms by which ICIs can cause irAEs. The first is cellular autoimmunity, which involves an increase in autoreactive T cell activity due to enhanced activation and cytotoxic function against antigens shared between the tumor and healthy tissue, as seen in conditions like myositis [21]. The second mechanism is humoral immunity. Although ICIs primarily function through T cell-mediated immunity, evidence suggests that B cells play a crucial role in both antitumor activity and irAEs. This occurs through increased levels of preexisting and/or new autoantibodies, which can be either tissue-specific or non-tissue-specific, leading to conditions such as thyroiditis, ICI-associated diabetes, bullous pemphigoid, and myasthenia gravis [20-21].

The third mechanism involves proinflammatory cytokines and chemokines. Reactive T cells secrete these molecules, creating a proinflammatory environment. The specific cytokines secreted appear to depend on the organ affected. For instance, IL-17 is associated with colitis, IL-1 and IL-6 with skin lesions, and IL-1B, IL-2, and GM-CSF with thyroiditis [21-22]. The fourth mechanism, specifically described in hypophysitis, involves complement cascade activation. This occurs when an anti-CTLA4 antibody directly binds to CTLA4 in healthy tissue, triggering the complement cascade [21].

Pathophysiology of ICI-Associated Pancreatitis

**Figure 3:**
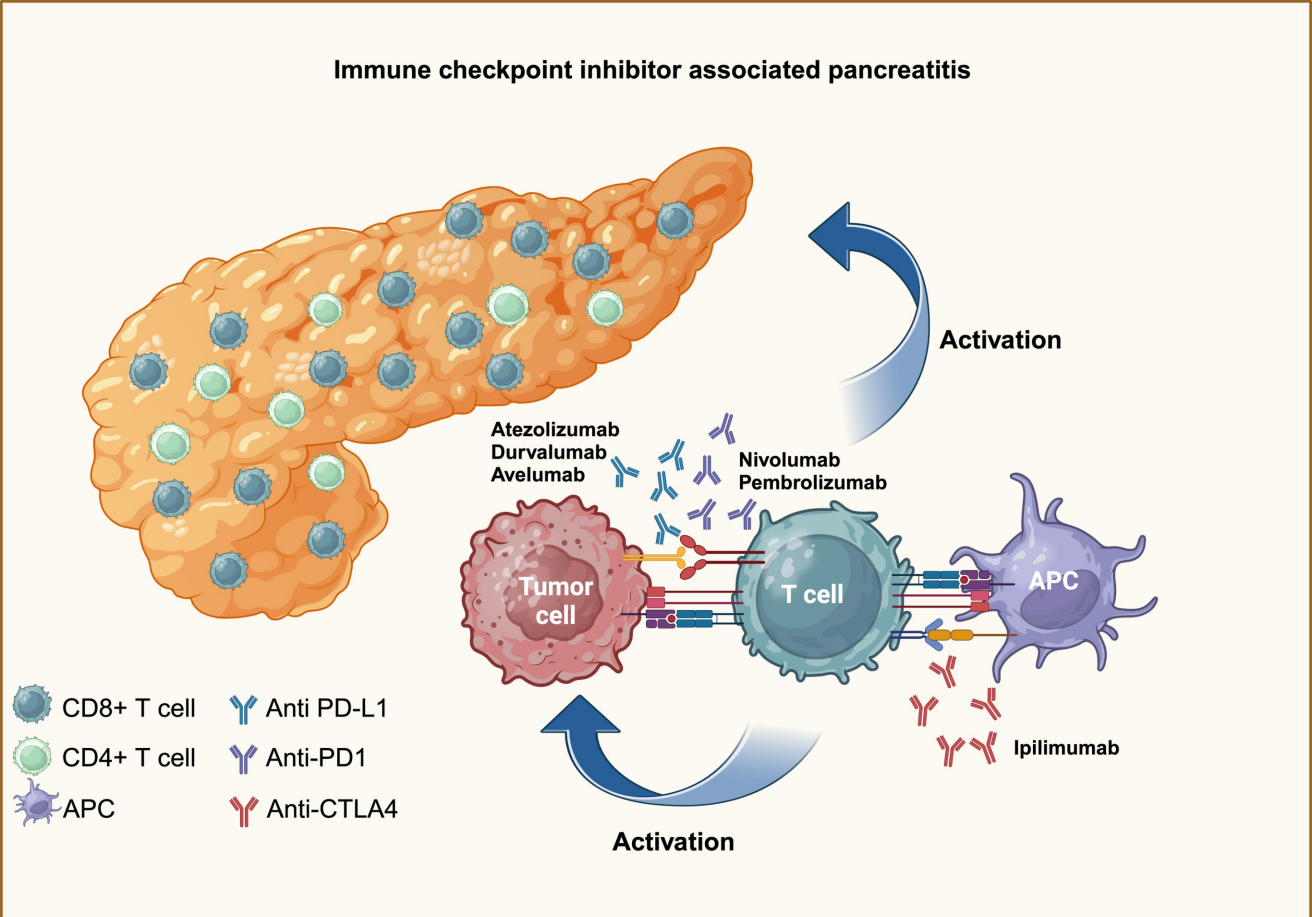
Infiltration of CD4+T cells and CD8+T cells into pancreatic tissue occurs due to the blockage of inhibitory signals on T cells by immune checkpoint inhibitors, resulting in damage to both exocrine and endocrine pancreatic tissue. Figure created in BioRender.com.

The pathophysiology of ICI-induced pancreatitis remains poorly understood. The current hypothesis suggests that the mechanism is primarily mediated by the infiltration of CD3+ T cells, particularly CD8+ T cells, into pancreatic tissue (Figure 3). This infiltration occurs due to the blockage of inhibitory signals on T cells by ICIs, resulting in damage to both exocrine and endocrine pancreatic tissue. Biopsied specimens have revealed infiltration of CD8+ T cells, T1A1+, and granzyme B+ lymphoid cells, supporting this hypothesis [6].

However, significant gaps remain in our understanding of the immunopathogenesis of ICI-mediated pancreatitis, warranting further investigation. Interestingly, studies have shown that slight elevations in pancreatic enzymes can occur in patients taking ICIs without accompanying clinical manifestations or radiographic abnormalities. This observation suggests that there is no clear association between enzyme elevation and the development of pancreatitis in these cases [23].

These findings highlight the complexity of diagnosing ICI-induced pancreatitis. Elevated pancreatic enzymes may be attributed to other factors, such as metastatic disease or renal failure, rather than pancreatic inflammation itself [6]. This underscores the need for a comprehensive approach to diagnosis, considering not only enzyme levels but also clinical presentation and imaging findings.

Incidence and epidemiology

Data on the incidence of pancreatitis in patients treated with ICIs vary across studies. A comprehensive review by Bouebaba et al. reported an overall incidence of ICI-induced pancreatitis of 1.9% [24]. However, individual studies have reported incidence rates ranging from 0.9% to 3.6% [25-26]. The variability in reported incidence may be attributed to differences in study populations, ICI regimens, and diagnostic criteria used for pancreatitis.

When comparing the incidence of pancreatitis in ICI therapy to other cancer treatments, ICIs generally show a higher risk. Traditional chemotherapy regimens have a reported pancreatitis incidence of 0.1-2% [27], while targeted therapies, such as tyrosine kinase inhibitors, have shown incidence rates of 0.5-2% [28]. However, it is important to note that the mechanisms of pancreatitis induction differ between these treatment modalities.

Several risk factors have been identified for ICI-induced pancreatitis. Combination ICI therapy, particularly the use of anti-CTLA-4 agents in combination with PD-1/PD-L1 inhibitors, has been associated with a higher incidence of pancreatitis compared to monotherapy [29]. A study by Brahmer et al. found that the incidence of grades 3-4 pancreatitis was 1.6% in patients receiving combination therapy, compared to 0.4% in those receiving PD-1 inhibitor monotherapy [30].

Patient demographics also play a role in the risk of ICI-induced pancreatitis. Age appears to be a factor, with some studies suggesting that older patients (>65 years) may be at higher risk [31]. However, this finding is not consistent across all studies, and further research is needed to clarify the impact of age on pancreatitis risk.

Pre-existing autoimmune conditions have been identified as a potential risk factor for ICI-induced irAEs, including pancreatitis [32]. Patients with a history of autoimmune disorders may be at increased risk of developing ICI-induced pancreatitis, although the exact magnitude of this risk remains unclear.

Tumor type may also influence the risk of ICI-induced pancreatitis. Some studies have suggested a higher incidence in patients with melanoma and renal cell carcinoma, but this may be confounded by the more frequent use of combination ICI therapy in these cancer types [33].

The timing of pancreatitis onset in ICI-treated patients is variable. While most cases occur within the first few months of treatment, late-onset cases have been reported, even after discontinuation of ICI therapy [34]. This highlights the need for ongoing vigilance throughout and beyond the treatment course.

Clinical presentation and diagnosis

Clinical Presentations of Immune Checkpoint-Associated Pancreatic Injury

The diagnosis of pancreatitis is confirmed when at least two of the following three criteria are met: 1) pain in the upper abdomen; 2) a threefold increase of lipase and amylase from the normal range; and 3) image findings consistent with acute pancreatitis [35]. ICI-induced pancreatitis could either be asymptomatic or symptomatic. Typical symptoms of acute pancreatitis are not common in this pathology as most patients have distinguishing clinical features. ICI-induced pancreatitis typically presents with a constellation of symptoms, including epigastric or diffuse abdominal pain, diarrhea, fever, and nausea [36]. Abdominal pain in the epigastric region is the most common symptom present in ICI-related pancreatitis [36]. The literature has reported that treatment of solid tumors induces adverse effects after several courses of this therapy. A case of an asymptomatic manifestation was observed in a middle-aged woman who was previously placed on combination therapy: ipilimumab and nivolumab. During therapy, the patient developed other presentations associated with endocrinopathies [37]. Pancreatitis was accidentally diagnosed through imaging techniques with features of inflammation. Table 1 provides further details on studies of asymptomatic patients who were diagnosed with pancreatitis associated with ICI therapy.

**Table 1:** Summary of studies of asymptomatic patients diagnosed with immune checkpoint inhibitor (ICI)-induced pancreatitis

Study	Study design	Results
Newman et al. [37]	Case report and review of literature	Middle-aged female patient with a history of malignant melanoma and on immunotherapy. Initial physical findings were unremarkable, and lab values were within normal limits. Six weeks post initiation of combination therapy, changes in endocrine tests were noted. Abdominal pain, elevated lipase, and amylase were absent in the patient. Characteristics of the inflamed pancreas were visible on the CT scan.
Friedman et al. [38]	Retrospective study	One hundred nineteen patients who had received the combination of ipilimumab at 3 mg/kg and nivolumab at 1 mg/kg were assessed to determine insult to the pancreatic tissues. Out of the 119 participants, 10 had a grade ≥3 amylase, 32 showed a grade ≥3 of lipase, and 10 had a grade ≥ 3 of amylase and lipase. Although this study was comprised mostly of asymptomatic manifestations of the disease, there were two cases in which patients presented with complaints. The first patient presented with fever, nausea, and vomiting a few days after completion of therapy, and pancreatic enzymes were not elevated, with no visible changes on image techniques. Nonetheless, the other patients’ complaints were abdominal pain, increased lipase, and amylase. Similarly, no changes were visible on the CT scan.

The symptomatology of immunotherapy-induced pancreatitis has been discussed in different studies. An elaborate study was carried out on different groups placed on PD-1 and CTLA-4 inhibitors with different dosages [38]. One group recorded 13% of patients with elevated lipase as the most common adverse event experienced with acceptable doses of combination therapy. The higher-dosed cohort had patients presenting with severely elevated lipase levels lasting over three weeks. As a result of this trial, the US Food and Drug Administration (FDA) approved 3 mg/kg ipilimumab plus 1 mg/kg nivolumab as a baseline therapy for melanoma [38]. These data prompted further research on the relationship between amylase, lipase, and ICI-related pancreatitis. A combination therapy of nivolumab (1 mg/kg) and ipilimumab (3 mg/kg) was administered to 119 participants [39]. The study reported that two patients developed pancreatitis. Additionally, 20% of patients exhibited elevated amylase levels; another 20% showed increases in both amylase and lipase, and 6.3% of patients presented with grade 3 or higher elevations in these enzymes. Similar results and clinical manifestations were consistent in other studies. In these studies, it is still unclear if changes leading to pancreatitis are directly induced by checkpoint inhibitors, as there are no substantial details to prove otherwise. Severe insult to the pancreatic tissues results in pancreatic insufficiency. This complication, observed in severe cases of immunotherapy-associated pancreatitis, leads to atrophy of the pancreas. The destruction of pancreatic cells leads to persistent symptoms, which can be alleviated through the administration of supplementary pancreatic enzymes. A case report previously reported a patient on treatment for a solid tumor with unremarkable physical findings [40]. Nonetheless, weeks later, the patient began passing loose stools with features of steatorrhea. Subsequently, blood samples were retrieved to rule out other etiologies, which revealed elevated lipase and decreased fecal elastase, concluding with exocrine pancreatic insufficiency as an adverse event of ICIs. This outcome accounts for approximately 1% of patients recorded with ICI-related pancreatitis, a phenomenon managed with replacement pancreatic enzymes [40]. Another case study highlighted the effect of anti-PD-1 antibody causing atrophic pancreatitis; lab values narrated declined fecal elastase, and abdominal diagnostic imaging was consistent with attributes seen in pancreatic atrophy [41]. However, it is uncertain if the synergistic effect of previously administered ipilimumab, in addition to the current therapy of pembrolizumab, led to the cause of atrophic exocrine insufficiency of the pancreas. The incidence of symptomatic presentations to the pancreas from immune-related therapy has been listed in Table 2.

**Table 2:** Summary of studies of patients with clinical manifestations of immune checkpoint inhibitor (ICI)-induced pancreatitis

Study	Study design	Results	Conclusion
Tan et al. [42]	Case series	A case series study was carried out using six patients with advanced carcinoma who received immune checkpoint inhibitors over two years. Over an average period of 105 days, features of pancreatic injury were visible in the patients. The average cycle was 4.5 (1-7) with either sequencing or concurrent therapy. Abdominal pain, elevated lipase, and amylase were eminent. Patients with a mild form of immune-associated pancreatitis had elevated amylase and lipase of 192 U/L and 3,135 U/L, respectively. The group with moderate staging had levels of 158 U/L and 1,179 U/L each. The most severe levels of amylase to lipase were 1,263 U/L and 14,970 U/L, respectively. These abnormalities persisted up until the 22nd week, but there was significant improvement and reversal of atypical values to normal with the use of steroids.	The study summarizes the progression of immune-related adverse events of the pancreas from mild to severe stages. Patients with severe forms showed a threefold increase in pancreatic enzymes. Among the different imaging techniques used, magnetic resonance cholangiopancreatography and magnetic resonance imaging were found to be the most ideal techniques for visualizing changes in the pancreas as the disease progressed from mild to severe.
Janssens et al. [43]	Case study	This study reports on a young woman with persistent abdominal pain in the epigastric region, elevated amylase at 136 U/L, and lipase at 439 U/L. Other blood tests carried out were within normal limits. At the onset of the disease, a CT scan visualized changes in the parenchyma of the pancreas but later detected enhancement in all parts of the pancreas with attributes of inflammation in the tissues. The patient was placed on prednisone therapy for 14 days, and symptoms were resolved.	This report highlights the lack of information regarding the management of immune checkpoint inhibitors-related pancreatitis, as well as the clinical manifestations of acute pancreatitis.
Rogers et al. [44]	Case study	A 55-year-old male with stage IIIB squamous cell lung cancer began treatment with durvalumab. Gradual increase in blood glucose levels within six months of durvalumab treatment, Elevated serum amylase and lipase levels (grade 2). Enzyme levels normalized within two weeks after discontinuing durvalumab. Treatment resumed and was completed without further complications.	ICI-associated pancreatitis can occur even without a history of pancreatitis. Temporary discontinuation of ICI treatment may allow for the resolution of symptoms and successful resumption of therapy.
Zhang et al. [45]	Retrospective cohort study	25% incidence of pancreatitis, markedly elevated blood glucose, low C-peptide levels, and discordance between high glucose and lower A1C.	ICIs can induce selective pancreatic endocrine or exocrine toxicity. Early identification and insulin treatment are crucial to avoid complications like DKA. Patient education on hyperglycemia symptoms is necessary.

Diagnostic criteria and imaging findings

Blood Screening

Enzymes and antibodies are indicators not to be dismissed when screening for ICI-induced pancreatitis. The most recurrent enzymes present in the pathology are amylase and lipase, which is a specification in the Common Terminology Criteria for Adverse Events (CTCAE) grading [36]. Lipase is considered a more effective marker, as they have a higher rate of sensitivity and specificity in diagnosing the adverse event [2 ]. Although this is considered an effective marker, elevated pancreatic enzymes without meeting the other criteria would not make an accurate diagnosis [36].

Imaging Technologies

Computed tomography (CT): This modality revealed inflammatory changes with edematous parts of the pancreas in a patient with unremarkable physical findings [37]. Hofmann et al. reported the swelling visible in the parenchyma of the pancreas and a decline in contrast uptake in tissues [36].

Magnetic resonance imaging (MRI): MRI detected the earliest abnormalities as the disease progressed. In mild to moderate forms, a decline in the intensity of signals and an increase in signals were noted, in addition to restricted diffusion. The most severe grade revealed morphological changes in the pancreas and a decrease in the intensification of signals [42]. This tool was declared effective in differentiating the inflammatory changes seen in different types of pancreatitis. Capurso et al. observed constraints in signals passing through heavier tissues observed using this modality [36].

Endoscopic ultrasound (EU): This device discovered hypoechoic tissues with enlarged parts of the pancreas associated with specifically “nivolumab pancreatitis” [43]. In cases of pembrolizumab-induced pancreatic adverse events, ultrasound imaging may reveal a heterogeneous pancreatic texture characterized by a mixture of hypoechoic and hyperechoic areas within the pancreatic tissue [46].

Endoscopic ultrasound-fine needle biopsy (EUS-FNB)/fine needle aspiration (EUS-FNA): In the absence of surgery, these are minimally invasive procedures that provide access to visceral organs to enable accurate diagnosis of histological cells. EUS-FNB discovered the prevalence of neutrophils with fibrosis, and igG4 plasma cells were not detected [43]. Several authors have highlighted the importance of EUS-FNA in diagnosing pembrolizumab-related pancreatitis compared with nivolumab-associated pancreatitis. Song et al. described the pancreatic ducts as being surrounded by lymphocytes and hardening of the stroma, which were noted using EUS-FNA in nivolumab-induced pancreatitis [47]. In pembrolizumab-induced pancreatitis, infiltration of T cells with CD8+ cells accounted for the majority of prevalent tissues over CD4+ cells [48].

Positron emission tomography-computed tomography (PET-CT): Janssens et al. reported heterogeneous enhancement in pancreatic parenchyma [43]. Some reports agree that PET-CT detected the saturation of 18F-fluorodeoxyglucose in pancreatic tissues [49-50].

Endoscopic retrograde cholangiopancreatography (ERCP): ERCP is not only used in biliary strictures but can also be used to detect abnormalities in ICIs-related pancreatitis. Tanaka et al. observed features similar to autoimmune pancreatitis (AIP), describing the narrowing of the pancreatic duct with the exemption of the main duct [51].

Differential Diagnosis

AIP is classified into type 1 AIP, which is associated with immunoglobulin G4 (igG4) and affects multiple organs, while type 2 only affects the pancreas. The relationship between AIP has been visible in radiological images. Although clinical manifestations may differ, a PET-CT scan detected similar features of both pathologies, notably enhanced areas in the pancreatic parenchyma [43]. Regarding acute interstitial pancreatitis, it is reported to be comparable to ICI-associated pancreatitis. The body and tail of the pancreas were significantly increased with fat strands surrounding the body. These characteristics are typically seen in acute interstitial pancreatitis [52].

Management and treatment

The CTCAE is widely used to grade the severity of cancer treatment-related adverse events. For ICI-associated pancreatitis, the incidence of grades 3 and 4 requiring medical intervention is estimated at 1%, while grades 3 to 5 occur in approximately 1.7% of cases [53].

**Figure 4:**
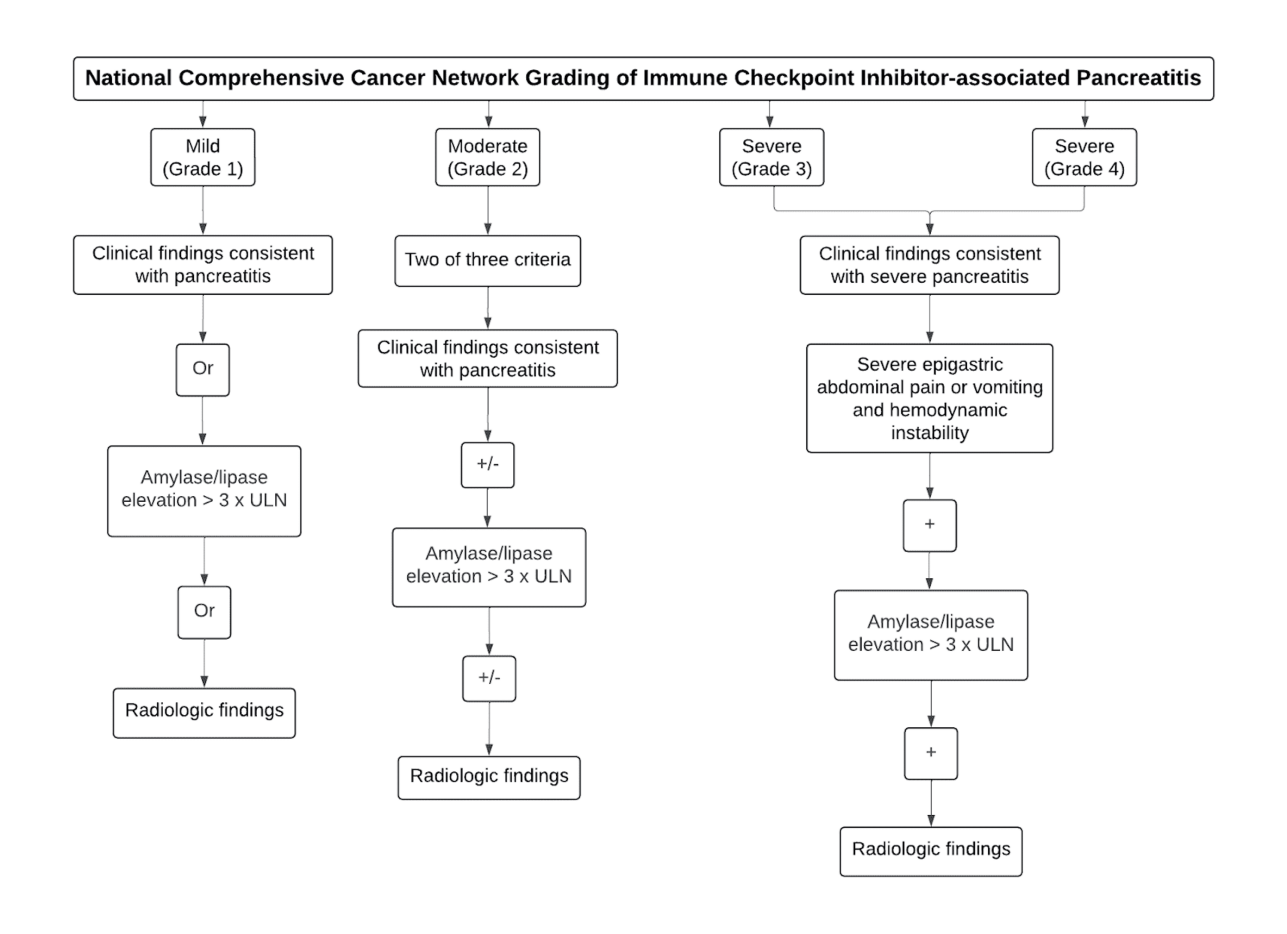
National Comprehensive Cancer Network (NCCN) grading system for immune checkpoint inhibitor-associated pancreatitis The flowchart categorizes the severity of pancreatitis into four grades: mild (grade 1), moderate (grade 2), and severe (grades 3 and 4). A combination of clinical symptoms, laboratory results, and imaging findings is utilized to determine the severity grade of ICI-associated pancreatitis, which is likely used to guide treatment decisions and patient management. The figure is created in BioRender.com.

The majority of cases present asymptomatic amylase/lipase elevation. Combination immunotherapy increases the incidence compared to single-agent regimens, with rates ranging from 6 to 10.60% [44,54]. The National Comprehensive Cancer Network (NCCN) guidelines recommend intervention for moderate to severe pancreatitis. Grading is based on amylase/lipase elevation, radiologic findings, and clinical symptoms [55] (Figure 4).

Management strategies for ICI-associated pancreatitis include the following:

1. IV fluid administration: Recommended within the first 48 hours for lipase elevation compatible with CTCAE grade 3 or higher. Controlled-balanced fluid replacement (5-10 mL/kg/hour or < 4.1 L over initial 24 hours) with lactated Ringer's solution is advised. IV fluids have been shown to decrease the risk of long-term sequelae related to immune-mediated pancreatitis [44,56].

2. Corticosteroid use: The efficacy of corticosteroids in ICI-associated pancreatitis is controversial. Some cases have required prednisone doses as high as 4 mg/kg/day with subsequent tapering over four to six weeks. However, Abu-Sbeih et al. demonstrated that corticosteroids do not improve overall survival or clinical outcomes regarding the prevention of short-term and long-term comorbidities [56-57].

3. Infliximab and tocilizumab: For steroid-refractory cases, infliximab (anti-TNFα) is recommended, with a potential repeated dose 14 days later. Tocilizumab (anti-IL-6) has been proposed for steroid-refractory irAEs, but further randomized trials are required to demonstrate its efficacy and safety in ICI-associated pancreatitis [55,58].

4. Antibiotic use: Routine administration of antibiotics is not recommended unless a concomitant infection is detected. Abu-Sbeih et al. reported that routine antibiotic use is not associated with better outcomes or increased survival [54].

5. Pain management: Appropriate use of analgesics is crucial for patient comfort. Options include acetaminophen, tramadol, and opioids, depending on pain severity [44].

6. Nutrition: Early enteral nutrition is recommended within the first 24-48 hours of hospitalization once the patient can tolerate oral feeding. This approach has been associated with a decreased incidence of infection, which can worsen prognosis and decrease survival rates [52,54].

7. Adjusting cancer treatment: Immediate discontinuation of ICI therapy is fundamental in managing pancreatitis. However, resumption of ICI has been associated with better outcomes and longer survival compared to permanent discontinuation despite an increased risk of relapse [54-56].

Risk stratification systems, such as the Whitlock Scoring System and the Bedside Index of Severity in Acute Pancreatitis (BISAP) score, can guide management decisions. The Whitlock system calculates readmission rates at discharge, while the BISAP index helps determine if ICU management is necessary and predicts mortality associated with pancreatitis [44] (Table 3). Long-term monitoring is essential, as ICI-associated pancreatitis can lead to chronic pancreatitis and secondary pancreatic insufficiency, both exocrine and endocrine [57].

**Table 3:** Bedside Index for Severity in Acute Pancreatitis (BISAP) score Adapted from Rogers et al. (2020). 0-2 points: low mortality (2%); 3-5 points: higher mortality (>15%).

Parameter	No	Yes
Blood urea nitrogen > 25 mg/dL	0	1
Altered mental status (Glasgow Coma Scale score < 15)	0	1
Systemic Inflammatory Response Syndrome (SIRS) ≥ 2 Criteria → Temp > 38°C (100.4°F) or < 36°C (96.8°F) → Heart rate > 90 beats per minute → Respiratory rate > 20 breaths per minute or arterial carbon dioxide tension (PaCO2) < 32 mmHg → WBC count > 12,000/uL OR < 4,000/uL OR > 10% immature (band) forms.	0	1
Age > 60 years	0	1
Pleural effusion present	0	1

Challenges and future directions

The exact mechanism of ICI-associated pancreatic injury remains poorly understood, contributing to the rarity of ICI-associated pancreatitis cases and hindering effective treatment strategies [6]. Additionally, tumor-inherent and treatment-related toxicities limit the clinical application of ICIs [13]. Despite some cases of long-lasting outcomes, most patients either show no response or develop resistance after initial tumor remission [59].

Further studies are crucial to advance this treatment modality. In-depth explorations of resistance mechanisms are needed to develop more targeted therapies and improve efficacy [59]. Research should also focus on the relationship between anti-tumor and anti-self-reactivity and the development of predictive biomarkers [60]. Systematic reviews and meta-analyses of emerging treatments and their clinical outcomes in pancreatic cancer-specific immunotherapy are essential [61].

To reduce the risk of pancreatitis, implementing appropriate protocols for irAE management with well-delineated mechanisms is crucial [6,60]. Comprehensive clinical evaluations before each immunotherapy dose are recommended. Furthermore, physicians must be aware of ICI-associated symptoms to effectively monitor patients [62]. These strategies, combined with ongoing research, will be vital in improving the safety and efficacy of ICI treatments while minimizing the risk of pancreatitis.

## Conclusions

ICIs have transformed cancer treatment but can cause irAEs, including pancreatitis. ICI-induced pancreatitis, though rare, presents challenges due to its variable presentation and potential for pancreatic dysfunction. Diagnosis involves clinical symptoms, elevated pancreatic enzymes, and imaging, with MRI and endoscopic ultrasound showing promise for early detection. Management includes IV fluids, pain control, and nutritional support, while the role of corticosteroids remains debated. Long-term monitoring is crucial due to the risks of chronic pancreatitis and pancreatic insufficiency. Future research should focus on understanding mechanisms, developing biomarkers, and refining treatment protocols to optimize patient care as ICI use expands.
